# Continuous-wave broadly tunable high-power Cr:CdS laser

**DOI:** 10.1007/s00340-014-5921-z

**Published:** 2014-09-14

**Authors:** E. Sorokin, D. Klimentov, M. P. Frolov, Yu. V. Korostelin, V. I. Kozlovsky, Yu. P. Podmar’kov, Ya. K. Skasyrsky, I. T. Sorokina

**Affiliations:** 1 Institut für Photonik, TU Wien, Gusshausstrasse 27/387, 1040 Vienna, Austria; 2 Department of Physics, Norwegian University of Science and Technology, 7491 Trondheim, Norway; 3 P.N. Lebedev Physics Institute, Russian Academy of Sciences, Leninsky prosp. 53, 119991 Moscow, Russia; 4 Moscow Institute of Physics and Technology (State University), Institutskii per. 9, Dolgoprudnyi, 141700 Moscow Region, Russia

## Abstract

We report spectroscopic characteristics and laser properties of the mid-infrared active laser medium Cr^2+^:CdS. Temperature-dependent absorption, luminescence and lifetime measurements of the ^5^E exited state allow determination of peak emission cross section value of 1.35 × 10^−18^ cm^2^ in *σ*-polarization at room temperature. Lifetime values vary from 7.6 µs at 8 K to 0.48 µs at 320 K, corresponding to 22 % quantum yield at 285 K. Under Tm-fiber laser pumping, the continuous-wave output reached 1.8 W at 2.5 μm with 35.5 % slope efficiency. With a single CaF_2_ prism, the CW Cr^2+^:CdS laser could be tuned from 2.240 to 3.285 µm.

## Introduction

The broadband mid-infrared laser media are of great interest for development of both widely tunable lasers with a narrow emission line and femtosecond lasers. Such lasers have potential applications in high-resolution spectroscopy, optical frequency metrology and science. Reaching mid-infrared wavelength region opens up additional application possibilities, such as trace gas analysis, remote sensing of the atmosphere, environmental monitoring, and medicine. For this reason, the relatively novel class of laser active media based on II–VI compounds doped with divalent transition metal ions [1, 2] attracts special attention. The II–VI compounds allow an important degree of flexibility by changing the chemical composition, which controls the absorption and emission spectral position via the unit cell and crystal field parameters [3].

From these media, the Cr^2+^-doped media are of special importance, because they allow efficient and high-power continuous-wave operation at or only slightly below room temperatures, thus eliminating the need for cryocooling and vacuum. Currently, the demonstrated chromium-doped II–VI compound lasers cover spectral range from 1.9 [4] to 3.6 µm [5]. Availability of high-quality ZnSe material, suitable for diffusion doping and having negligible thermal quenching well above room temperature, enabled a number of successes to be demonstrated with Cr:ZnSe material. These include ultrabroad tuning range between 1.880 and 3.349 µm [4, 6] and mid-IR femtosecond lasers [7], and a variety of applications of Cr^2+^:ZnSe laser in particular for high sensitivity and high-resolution spectroscopy [8–16] have been demonstrated. In the recent times, Cr:ZnS material has also become widely used. Its main advantage of excellent thermo-optical quality outweighs the somewhat stronger thermal quenching, making it a material of choice for high-power systems, continuous-wave [17] as well as femtosecond [18, 19].

Extension of the operation wavelength of Cr-doped materials further into the mid-infrared region requires using hosts with lower crystalline field, such as Cd-based chalcogenides [3, 20–22]. On this way, it has been shown that Cr^2+^:CdSe laser could be tuned farther than 3.35 μm [23, 24]; however, another member of the II–VI compounds family Cr^2+^:CdS has yet remained barely studied due to the unavailability of the good optical quality single crystals. Having similar to Cr^2+^:CdSe spectroscopic properties, Cr^2+^:CdS crystal is known to have the larger bandgap, the better hardness and thermo-optics, and much higher thermal conductivity (15 vs. 6.5 W/mK in Cr:CdSe [3]). In analogy to the Cr:ZnSe vs. Cr:ZnS comparison, we expect the power handling capability of this material to exceed that of Cr^2+^:CdSe. The analogy is not complete, as both Zn-based hosts prefer the cubic phase, while CdS and CdSe crystallize in the wurtzite structure. Therefore, the properties of the CdS material have to be correctly assessed in comparison to the already known active media.

Previous study [25] focused on room-temperature spectroscopic properties of Cr^2+^:CdS and on properties relevant to the pulsed laser performance. The initial results on continuous-wave lasing with 0.8 W output power at 2.534 μm have been reported recently [26]. Spectral tuning range of CW Cr^2+^:CdS has not been studied.

In this paper, we report the first continuous-wave broadly tunable around-room-temperature operation of Cr^2+^:CdS pumped by the Tm-fiber laser at 1.91 μm. The laser yields up to 1.8 W output power and is tunable over 1,000 nm from 2.240 to 3.285 µm. We also present the results of extensive spectroscopic study, including temperature-dependent lifetime measurements, fluorescence and absorption spectra, and provide the values for absorption and emission cross sections and saturation intensity.

## Preparation of samples

In this study, we used the seeded physical vapor transport technique in vertical configuration [27] to grow CdS crystal doped with chromium at a concentration of 1.1 × 10^18^ cm^−3^, which was measured by atomic emission analysis. The Cr^2+^:CdS boule grown on a single-crystal seed at 1,100–1,250 °C temperature was about 1 cm long and 3 cm in diameter. The vapor-phase mass transfer was performed by a physical transport in helium. Homogeneous chromium doping was achieved straightforwardly during the growth process by using the technology developed earlier for growing highly perfect, optically homogeneous solid–solution single crystals [28]. Typical passive losses in this material did not exceed 0.026 cm^−1^ at the lasing wavelengths [29].

Two samples in the form of plane-parallel plate were fabricated from the boule. For spectroscopic measurements, we used a ~5 × 5 × 5 mm sample, oriented to allow recording of absorption and emission spectra in both *π* and *σ* polarizations. For laser experiments, we used a 5-mm-long sample with the *c*-axis nearly perpendicular to the polished faces. The uncoated optical surfaces of both samples were mechanically polished to an optical finish and were parallel to each other with an accuracy of 30 arc seconds.

## Spectroscopic studies

**Fig. 1:**
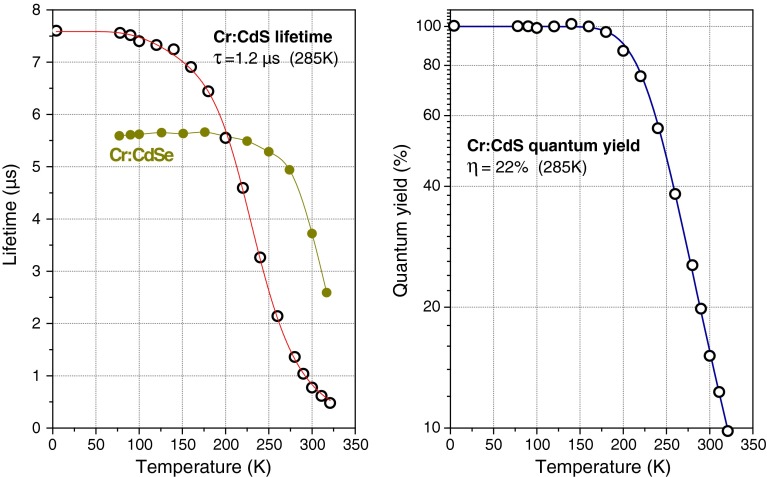
*Left graph* Fluorescence lifetime in Cr:CdS (*open dots*). The *red line* is a fit to the lifetime dependence using a phonon-assisted single activation energy model [30]. *Closed dots* show the lifetime of the Cr:CdSe crystal for comparison (slight lifetime increase at 100–175 K is due to the reabsorption in the high-concentration Cr:CdSe sample). *Right graph* Fluorescence quantum yield in Cr:CdS crystal. The *blue line* is the data fit using the above model. For better resolution around room temperature, the data and fit are plotted in the semi-log scale

Temperature-dependent photoluminescence lifetime measurements of the upper laser ^5^E level of Cr^2+^ in single-crystal CdS were performed using pulsed excitation with a 0.5 W laser diode at 1.6 µm. A variable-temperature optical cryostat allowed measuring the lifetime between 8 and 320 K. The emission lifetime (open dots in Fig. 1a) shows a temperature dependence, which is typical for the vibronic media. It is well approximated by a phonon-assisted, single activation energy model [30] (red line in Fig. 1a) with $\hbar \omega \approx 340$ cm^−1^ and $E_{\text{act}} = 1,660 \,{\text{cm}}^{ - 1}$. At room temperature, the lifetime is strongly quenched (emission lifetime is 1.2 µs at 285 K). Dividing the lifetime data by the radiative phonon-assisted emission rate allows calculating the luminescence quantum yield (Fig. 1b), which is about 22 % at 285 K. In comparison, the Cr:CdSe crystal (filled dots in Fig. 1) exhibits shorter radiative lifetime, much lower lifetime quenching at room temperature, but somewhat sharper decrease at higher temperatures. This behavior strongly resembles the lifetime dependences of Cr:ZnS and Cr:ZnSe, respectively.

To record the infrared absorption spectra, we used a double-beam infrared spectrophotometer, inserting the cryostat and a polarizer into the sample compartment. Absolute values of absorption cross sections were calculated from absorption spectra assuming that all chromium ions (1.1 × 10^18^ cm^−3^) are in the Cr^2+^ charge state.

Photoluminescence spectra measurements under Er:fiber laser excitation at 1.6 µm were performed using a Fourier transform spectrometer with a cooled InSb detector and corrected for the response of the detection system using a calibrated tungsten band lamp. Using the radiative lifetime data, it is possible to calculate the emission cross section from the fluorescence intensity signal including the polarization components [31].

**Fig. 2:**
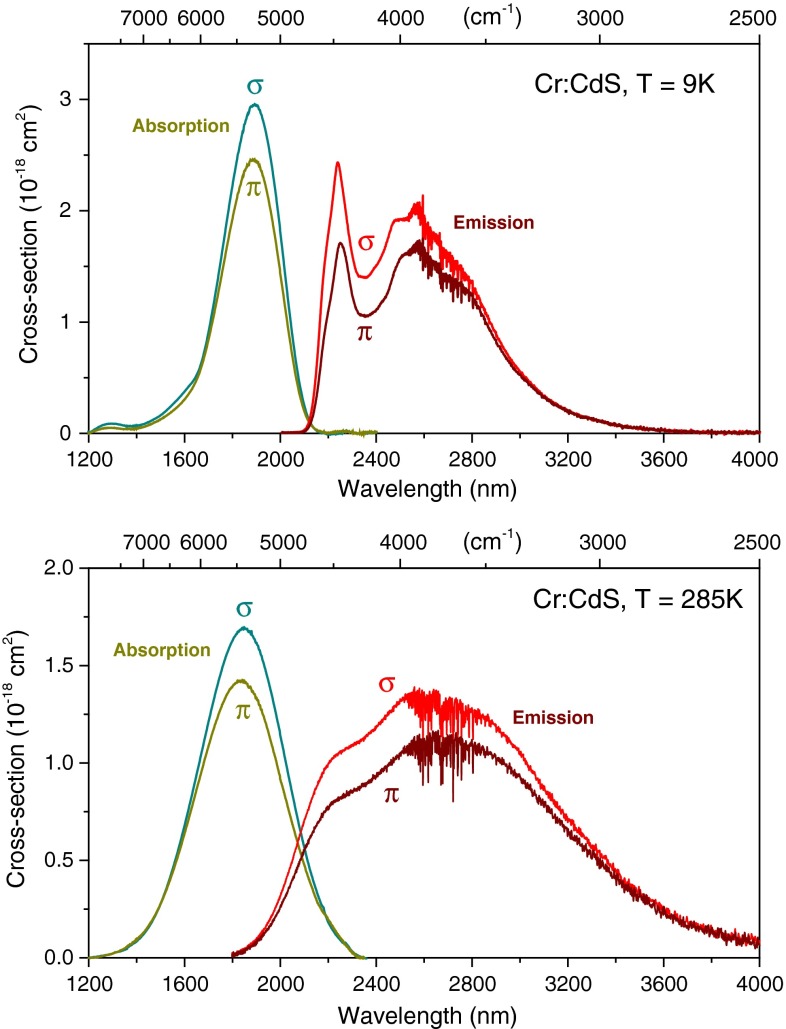
Polarized absorption and emission spectra of Cr:CdS at 9 and 285 K

Figure 2 shows absorption and emission (gain) cross section spectra of Cr^2+^:CdS recorded at 9 and 285 K. The high-frequency noise in the spectra around 2.7 µm is due to the atmospheric water vapor absorption, not completely eliminated in the correction. The room-temperature infrared emission of Cr^2+^:CdS centers at ~2.6 µm with a bandwidth of 1,100 nm full-width at half-maximum and extends to much longer wavelengths than that of Cr^2+^:ZnSe, giving the possibility of extended tuning in the mid-infrared well above 3 µm. It is interesting to note that *σ*-polarization is 15–20 % stronger than *π*-polarization for both absorption and emission and should be preferred in laser design.

## Laser experiments

**Fig. 3:**
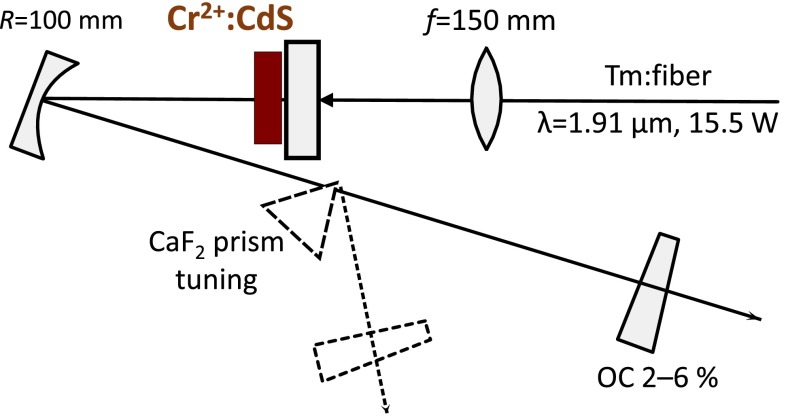
Schematic of the laser experimental setup. The output radiation is polarized in the plane of the figure. *OC* output coupler, *f* focusing lens

**Fig. 4:**
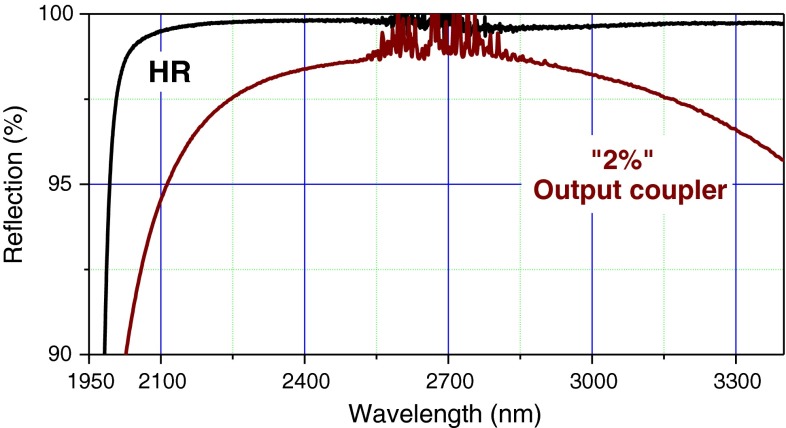
Reflectivity curves of the high reflector (HR) and the broadband output coupler, used in the tuning experiments. The high-frequency modulation at 2.6–2.8 µm is caused by the incomplete cancellation of the water absorption lines in the spectrophotometer

Figure 3 shows the experimental setup for the laser performance studies. The laser cavity is formed by a flat dichroic high reflector with very high reflectivity for the laser wavelengths and nearly transparent at pump wavelength (transmission 92 %), a folding mirror with *R* = 100 mm radius of curvature, and the plane output coupler, varying between 2 % for tuning experiments and 6 % for high-power generation. The optical coatings have been manufactured in the hybrid semiconductor oxide technology, allowing for a very high index contrast and large bandwidth. With a design center wavelength at 2.6 μm, the useful bandwidth of high-reflecting mirrors covered the 2–3.5 µm range and 2.2–3.3 µm for the output coupler at 3 % transmission level (Fig. 4). The 6 % output coupler was centered at 2,450 nm.

The Cr^2+^:CdS laser crystal, absorbing 46 % of light at 1.91 μm, was longitudinally pumped through the high reflector. The laser crystal was side-mounted in a water-cooled heat sink, with the *c*-axis approximately along the laser beam propagation direction, so that the active crystal has been operated in *σ*-polarization for both laser and pump radiation. The unpolarized Tm-fiber laser from IPG Photonics, operating at up to 15.5 W output power at 1.91 μm, was focused onto the crystal by the 150-mm lens into the spot size smaller than 60 μm. For wavelength tuning experiments, a Brewster cut CaF_2_ prism was inserted in the modified cavity. The laser wavelength was measured using a FTIR spectrometer and output power by a thermopile power meter. All experiments were performed at the heat sink temperature of 285–287 K.

**Fig. 5:**
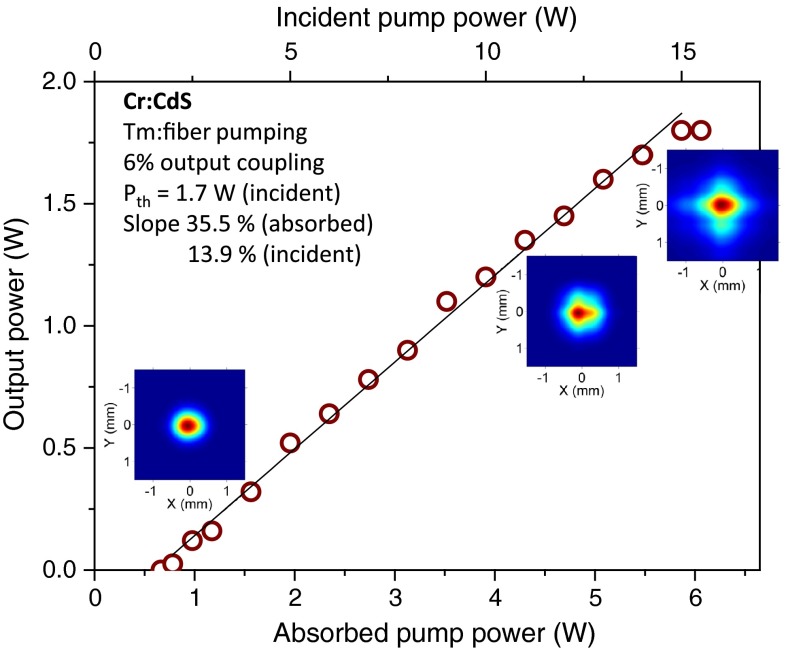
Output power of a continuous-wave Cr:CdS laser. The *color insets* show the mode intensity distribution at low, medium and high power

Figure 5 shows the laser output characteristics measured with 6 % output coupler. Without wavelength selecting elements and purging, the laser operates between 2,540 and 2,555 nm. The laser output reached ~1.8 W at 6 W of absorbed power. To avoid the high reflector damage, the pump focus was moved away from the mirror. We measured slope efficiency of ~14 %, limited by the losses at the input face of the crystal (Fresnel reflection coefficient 15.2 %) and weak absorption of pump radiation by the crystal (46 %). With respect to the absorbed pump power, the slope efficiency reached 35.5 %. Even without polarizing elements in the cavity, the output radiation is mostly linearly polarized due to the slight misorientation of the *c*-axis. By rotating the sample, we made sure that this intrinsic polarization lies in the plane of Fig. 2. The mode profiles at the laser output (colored insets in Fig. 4) demonstrate building up of a cross-like shape at high power, typical for the induced birefringence influence. This is caused by the orientation of the active medium with the optical axis along the bam propagation direction. Orienting the optical axis perpendicular to the beam direction would allow using the intrinsic birefringence to suppress the thermal depolarization, and improve both the mode quality and efficiency.

With 6 % output coupling, we have measured threshold to be about 600 mW of absorbed pump power, compared with ~200 mW for Cr:CdSe in similar cavity [5]. This is obviously due to the strong lifetime quenching at room temperature (Fig. 1). However, once a threshold is reached, the high thermal conductivity of Cr:CdS allows easy power scaling, and we anticipate that it would surpass Cr:CdSe in this respect, in analogy to Cr:ZnS vs. Cr:ZnSe active media [17].

**Fig. 6:**
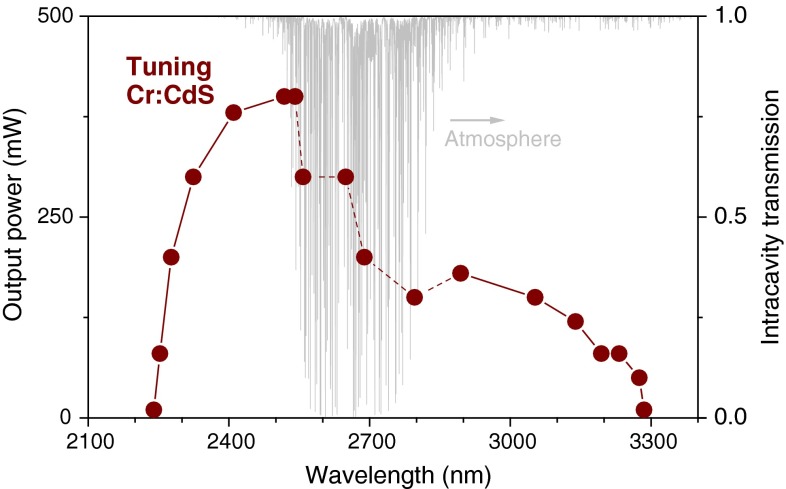
The tuning curve of a continuous-wave Cr^2+^:CdS laser recorded at 8 W pump power. The *gray spectrum* represents the transmission spectrum of atmosphere normalized to the round-trip cavity length at ~50 % relative humidity

The tuning experiments were performed in open air, with relative humidity 35–55 %. Using an intracavity CaF_2_ prism, we were able to demonstrate tunability over ~1,000 nm from 2.240 to 3.285 μm (Fig. 5) at 10 mW level. To achieve the broad tuning range, it is important to reduce the threshold as much as possible. We used a low-transmission output coupler (Fig. 4) and optimal pump focusing at the crystal entrance face. The incident pump power was kept at 8-W level to avoid the high reflector damage. The tuning range has characteristic dips around 2.7 μm due to water vapor absorption in the cavity (gray spectrum in Fig. 6). In the 2.5–2.8 μm spectral range, the laser would tune only by switching frequencies to positions between the water absorption lines. To avoid this, dry air purging or complete evacuation of the laser cavity would be required.

On the blue side, the tuning range is limited by the Cr^2+^:CdS self-absorption, while on the red side, the tuning range is limited only by the intracavity losses—the reflection on uncoated surfaces and increasing transmission of the output coupler. We expect that by using an optimized output coupler (with smaller transmission on the long-wavelength side) and antireflection coating, the tuning could be extended way beyond the demonstrated 3.3 µm, which is very interesting for possible spectroscopic applications.

## Conclusion

Summarizing, we report the first CW tuning at the highest reported so far output power of up to 1.8 W and the results of the extensive spectroscopic and laser analysis of Cr^2+^:CdS crystals. With the progress in crystal growth and optical quality of the material, ultrabroadband tunable and high-power laser operation and possibility of further extension of the tuning range in this alternative to Cr^2+^:ZnSe and Cr^2+^:CdSe medium up to 3.5 μm will become possible. The laser efficiency can be increased by optimization of the output coupling transmission and better matching the pump and lasing regions in the active element. To improve efficiency, one should opt for the directly mirror-coated or a Brewster-angled active element with a better absorption at the pump wavelength by using longer samples or higher Cr doping. The reported results demonstrate already now a potential of Cr^2+^:CdS for compact high-power tunable mid-infrared laser sources.
